# Chicken IgY Fc expressed by *Eimeria mitis* enhances the immunogenicity of *E. mitis*

**DOI:** 10.1186/s13071-016-1451-3

**Published:** 2016-03-21

**Authors:** Mei Qin, Xinming Tang, Guangwen Yin, Xianyong Liu, Jingxia Suo, Geru Tao, Saeed EI-Ashram, Yuan Li, Xun Suo

**Affiliations:** State Key Laboratory of Agrobiotechnology, College of Veterinary Medicine, China Agricultural University, Beijing, 100193 China; National Animal Protozoa Laboratory & College of Veterinary Medicine, China Agricultural University, Beijing, 100193 China; Key Laboratory of Animal Epidemiology and Zoonosis of Ministry of Agriculture, China Agricultural University, Beijing, 100193 China; Engineering Laboratory of Animal Pharmaceuticals, College of Animal Science, Fujian Agriculture and Forestry University, Fuzhou, Fujian Province 350002 China

**Keywords:** *Eimeria mitis*, Stable transfection, Chicken IgY Fc, Protective immune response

## Abstract

**Background:**

*Eimeria* species are obligate intracellular apicomplexan parasites, causing great economic losses in the poultry industry. Currently wild-and attenuated- type anticoccidial vaccines are used to control coccidiosis. However, their use in fast growing broilers is limited by vaccination side effects caused by medium and/or low immunogenic *Eimeria* spp. There is, therefore, a need for a vaccine with high immunogenicity for broilers.

**Methods:**

The avian yolk sac IgY Fc is the avian counterpart of the mammalian IgG Fc, which enhances immunogenicity of Fc-fusion proteins. Here, we developed a stable transgenic *Eimeria mitis* expressing IgY Fc (Emi.chFc) and investigated whether the avian IgY Fc fragment enhances the immunogenicity of *E. mitis*. Two-week-old broilers were immunized with either Emi.chFc or wild type *Eimeria* and challenged with wild type *E. mitis* to analyze the protective properties of transgenic Emi.chFc.

**Results:**

Chickens immunized with Emi.chFc had significantly lower oocyst output, in comparison with PBS, mock control (transgenic *E. mitis* expressing HA1 from H9N2 avian influenza virus) and wildtype *E. mitis* immunized groups after challenge, indicating that IgY Fc enhanced the immunogenicity of *E. mitis*.

**Conclusions:**

Our findings suggest that IgY Fc-expressing *Eimeria* may be a better coccidiosis vaccine, and transgenic *Eimeria* expressing Fc-fused exogenous antigens may be used as a novel vaccine-delivery vehicle against a wide variety of pathogens.

## Background

Avian coccidiosis, an intestinal disease caused by apicomplexan parasites of the genus *Eimeri*a, causes significant economic losses to the poultry industry throughout the world [1]. Coccidiosis can be effectively controlled by chemical pharmaceuticals. However, chemical treatment or prevention is associated with drug residues in poultry products and leads to drug resistance by the parasites [2–4]. Several *Eimeria* vaccines, such as Coccivac®, Immunocox®, Paracox® and Livacox® have been used successfully for the control of coccidiosis in breeders and layers [1, 5]. However, vaccines have not been widely used in broilers due to vaccination side effects and relatively low immunogenicity [6–8]. Host immunity to avian coccidiosis is complex, predominantly cell-mediated and requires at least two to three times of reinfection after vaccination with medium or low immunogenic *Eimeria* spp. [1, 9]. There is, therefore, a need for a highly effective vaccine which can elicit enhanced immune response to *Eimeria* to prevent coccidiosis for broilers.

The neonatal Fc receptor (FcRn) can transport IgG antibody across mucosal surfaces [10–12] and the FcRn-mediated transport was used to enhance the immunogenicity of Fc-expressing plasmid DNA and Fc-fusion proteins in mammals [12, 13]. Previous research revealed that the avian IgY Fc receptor (FcRY) exhibits the same binding character to IgY as the mammalian FcRn [14, 15]. We hypothesize that the chicken IgY Fc fragment facilitates the uptake of *Eimeira *antigen leading to enhanced immune responses against *Eimeria*. Importantly, transient and stable transfection of *Eimeira mitis* are well established [16]. In the present study, we developed transgenic *Eimeria mitis* expressing IgY Fc (Emi.chFc) and showed that broilers immunized with Emi.chFc elicited higher protective immune response against wild-type *E. mitis* than wild type *E. mitis*-immunized chickens.

## Methods

### Ethics statement

All animal research was approved by the Beijing Association for Science and Technology (approval ID SYXK (Beijing) 2007–0023) and was in compliance with Beijing Laboratory Animal Welfare and Ethics guidelines as issued by the Beijing Administration Committee of Laboratory Animals. All animal studies were also performed in accordance with the China Agricultural University Institutional Animal Care and Use Committee guidelines (ID:SKLAB-B-2010-003) and approved by the animal welfare committee of China Agricultural University.

### Parasites, chickens and cell culture

Oocysts of *E. mitis* (Zhuozhou strain) were propagated according to established protocols [17]. The transgenic *E. mitis* expressing HA1 region from H9N2 avian influenza virus (Emi.HA1) used as a mock control for IgY Fc transgenic *E. mitis* (see below) in the study was well established in our laboratory (unpublished data).

One-day-old Arbor Acre (AA) broiler chickens were purchased from Beijing Arbor Acres Poultry Breeding Co., Ltd. They were housed in isolators and fed with pathogen-free diet and water.

Madin-Darby bovine kidney (MDBK) cells were cultured in DMEM medium supplemented with fetal bovine serum (10 % v/v) and 1000 U penicillin/streptomycin in a humidified atmosphere with 5 % CO_2_ at 37 °C.

### Plasmid construction and transfection of *E. mitis*

Chicken IgY Fc fragment containing the CH2, CH3 and CH4 domains (GenBank: X07174) was synthesized by Aoke Peak Biological Science and Technology Co.Ltd. (China, Beijing) after codon optimization.

**Fig. 1:**
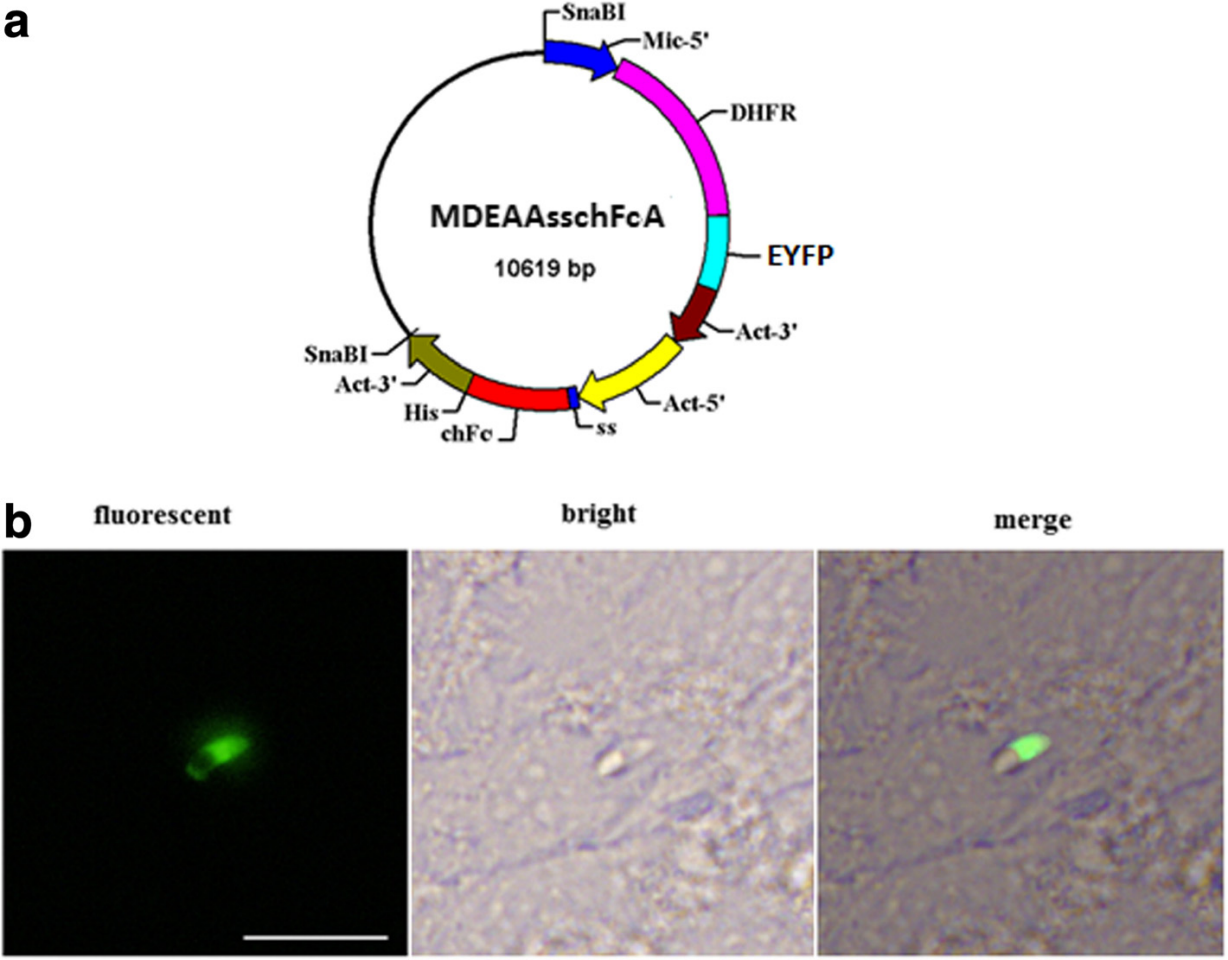
Schematic representation of EYFP containing plasmid and transient expression after inoculation in MDBK cells. **a** Expression cassettes are colored. The EYFP coding region is flanked by the Mic2 promoter and 3’region of actin from *E.tenella*. The foreign protein region is flanked by the promoter of actin (Act), 3’region of actin and signal sequence (ss,85 bp) derived from dense granule protein 8 (GRA8) of *Toxoplasma gondii.* **b** Sporozoites transfected with theMDEAAsschFcA plasmid and grown in MDBK cells. *Scale-bar*: 20 μm

The double expression-cassette plasmid, Mic-DHFR-EYFP/ACT-chFc-ACT (pMDEAAsschFcA) was constructed based on the pMic-EYFP/ACT-RFP plasmid as previously reported [18], with the RFP and EYFP genes replaced by chFc (Primer P1/P2) and TgDHFR-EYFP (Primer P3/P4) genes, respectively (Fig. 1a and Table 1). With the help of the integrated pyrimethamine resistance gene DHFR-TSm2m3, highly effective fluorescent oocysts were obtained [16, 19]. The plasmid DNA was linearized by SnaBI restriction enzyme to release the two expression cassettes from the backbone of the plasmid.

**Table 1:** Primers used in PCR

Primer	Forward primer	Reverse primer
P1/P2	5’ *CCTAGG*CAGGCAGGCAAGCCTAGGGC	5’*CCGCGG*TTAGTGGTGGTGGTGG
	TGCGGGAACAGCCAGT3’	TGGTGCGCGACACAAG3’
P3/P4	5’*GGTACC*ATGCAGAAGCCGGTGTGT	5’*CCTAGG*AAGCTTCTTGTACAGCT
	CTGGT3’	CGTCC3’
P5/P6	5’ATGGTGAGCAAGGGCGAGGA3’	5’AAGCTTCTTGTACAGCTCGT3’
SP1	_	5’AAGGCGAGCGAAGCTGTTCACT3’
SP2	_	5’CCATGCTTGGAGGAAACTTTGC3’
SP3	_	5’GCCTCTCGAAGGATCTGAATGC3’

For plasmid transfection, 10^7^ sporozoites freshly purified through a DE-52 cellulose column, 10 μg linearized DNA plasmid and 5 μl SnaBI were subjected to Nucleofector transfection (Program U-033, AMAXA, Switzerland). Half of the transfected sporozoites were examined for transient EYFP expression after inoculation onto confluent MDBK cells. The other half of the transfected sporozoites were inoculated into 7-day-old chickens *via* the cloacal route. The oocysts in fecal samples at 5–8 days post-inoculation were collected, purified and examined by fluorescence microscopy (Leica, Germany) [18, 20].

The transfected oocysts expressing EYFP (Emi.chFc) were selected in chickens by pyrimethamine (Sigma-Aldrich Co., St. Louis, Mo., USA) supplemented in feed and the MoFlo® Cell Sorter (Dako-Cytomation, Fort Collins, CO) on the single-cell mode, and then inoculated into coccidian-free chickens for the propagation of next generation [20]. The purified oocysts were re-suspended in 2.5 % KCr_2_O_7_ and stored at 4 °C for further experiments.

### Genomic DNA analysis of transgenic *E. mitis*

Genomic DNA from Emi.chFc was prepared as previously described [21]. PCR primer pairs (forward and reverse primer sequences) targeting the chFc and YFP genes were P1/P2 and P5/P6, respectively (Table 1). The chicken Fc and YFP genes were amplified employing the above total genomic DNA as templates. No template and mock-transfected parasite DNA samples were served as negative controls and plasmid MDEAAsschFcA was used as a positive control.

A genome walking kit (Takara, Dalian, China) was exploited to detect whether the plasmid was integrated into the *E. mitis* genome. According to the sequence of the MIC2 promoter, specific reverse primers were designed, SP1, SP2 and SP3 (Table 1). The forward primers AP1, AP2, AP3 and AP4 were supplied in the Genome Walking Kit. The following PCR amplification conditions were used: First-round PCR reaction: 94 °C for 1 min, 98 °C for 1 min, 94 °C for 30 s, 65 °C for 1 min, and 72 °C for 2 min for 5 cycles; 94 °C for 30 s, 25 °C for 3 min, and 72 °C for 2 min; 94 °C for 30 s, 65 °C for 1 min, and 72 °C for 2 min; 94°C for 30 s, 65 °C for 1 min, and 72 °C for 2 min for 15 cycles; 94 °C for 30 s, 44 °C for 1 min,72 °C for 2 min, and 72 °C for 10 min. The second- and third-PCR reactions consisted of 94°C for 30 s, 65 °C for 1 min, and 72 °C for 2 min; 94 °C for 30 s, 65 °C for 1 min, and 72 °C for 2 min; 94 °C for 30 s, 44 °C for 1 min, and 72 °C for 2 min for 15 cycles; and an extension at 72 °C for 10 min. The third-round PCR products were selected, purified and cloned into pEASY-T1-simple vector (TransGen Biotech, China) and confirmed by DNA sequencing. The resulting sequences were then analyzed by DNAStar7.0 software, and the integration sites in the genome were identified by performing a BLAST search in the *E. mitis* DB database.

### Western blot analysis

Protein extraction from Emi.chFc was carried out as previously described [21]. The total soluble protein from transgenic *E. mitis* was resolved by sodium dodecyl sulfate-polyacrylamide gel electrophoresis (SDS-PAGE) and electro-transferred onto a polyvinylidene difluoride (PVDF) membrane. For the detection of the target chFc protein, the membrane was probed with HRP-conjugated goat anti-chicken IgY Fc directly as primary antibodies.

### Indirect immunofluorescence assay

The indirect immunofluorescence assay was used to localize the foreign chicken Fc protein in *E. mitis*. Sporozoites purified by DE-52 anion-exchange were applied onto a poly-L-lysine-coated slide, followed by incubation in acetone at –20 °C for 20 min. After washing 3 times with PBS, the samples were permeabilized by incubation with Triton X-100 (0.1 % in PBS) for 15 min. To prevent unspecific binding of the detection antibody, the samples were blocked with 1 % BSA in PBS for 60 min. This was followed by 60 min incubation with Cy3-conjuated goat anti-chicken IgY Fc fragment (Jackson). The transgenic *E. mitis* sporozoites were examined under a laser scanning confocal microscope (SP5, Leica, Germany).

### Immunogenicity assay

Two-week-old AA broilers were orally vaccinated with 1000 freshly sporulated Emi.chFc, 1000 freshly sporulated wild-type (WT) oocysts of *E. mitis,* or 1000 freshly sporulated Emi.HA1 (as the mock control) (*n* = 6). An unvaccinated control group was orally given PBS. Fourteen days after vaccination, all groups were challenged with 4 × 10^5^ wild type sporulated oocysts of *E. mitis*. Oocysts shedding per bird was determined 5–8 d after the vaccination and challenge infection using the McMaster egg counting chamber [21, 22].

### Statistical analysis

Statistical analysis was performed using one-way ANOVA in the IBM SPSS Statistics 20 for Windows software. Differences between groups with a *P-*value of < 0.05 were considered to be statistically significant.

## Results

### chFc - *E. mitis* transfection and YFP expression

*E. mitis* sporozoites were transfected with the plasmid MDEAAsschFcA expressing YFP protein. EYFP expression was observed in transgenic sporozoites (Fig. 1b).

**Fig. 2:**
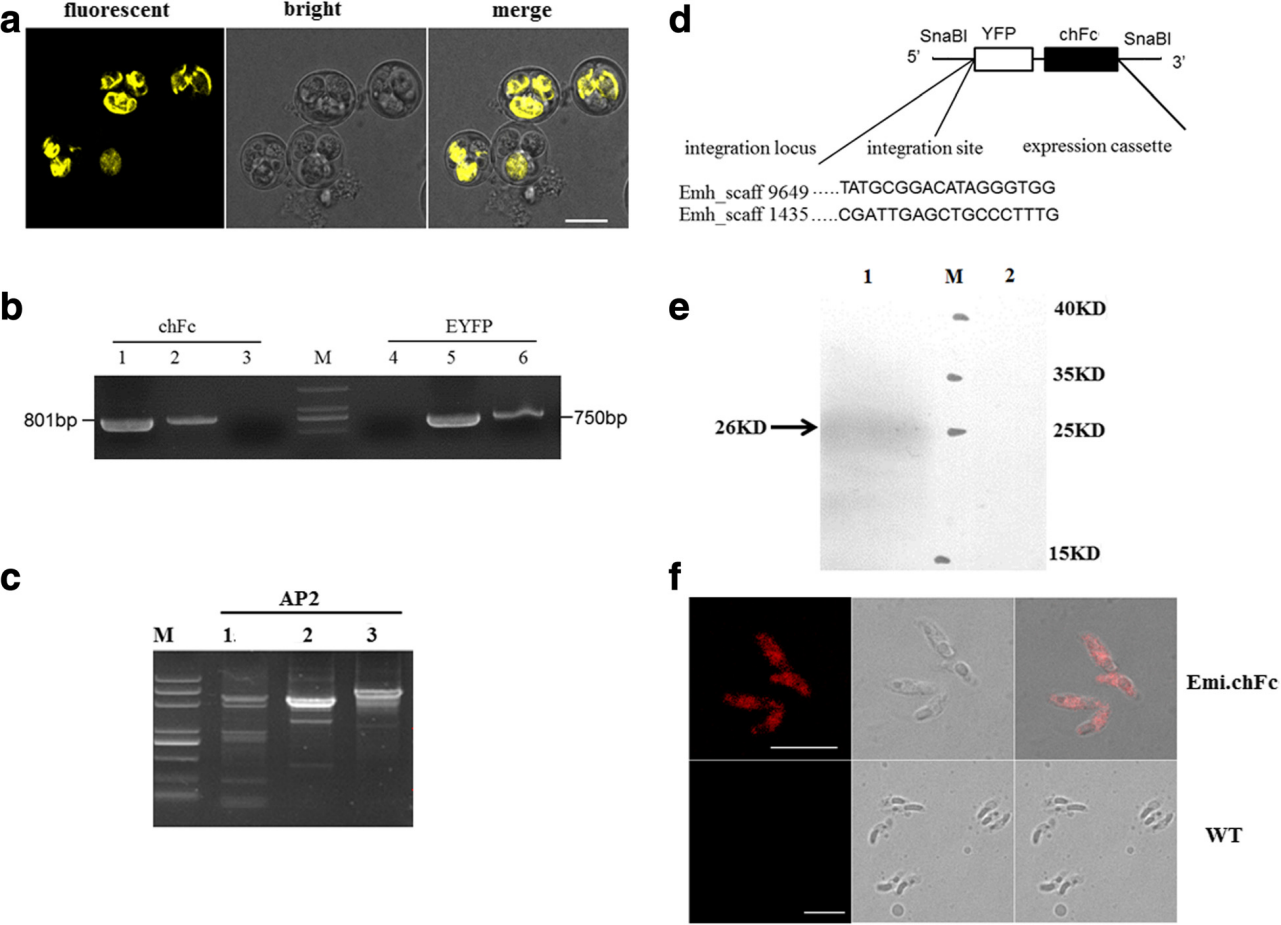
Verification of stable Emi.chFc population. **a** Fluorescent images of the fifth generation of Emi.chFc oocysts. **b** Detection of chFc and YFP genes by conventional PCR. Lanes 1 and 5, PCR products from plasmid pMDEAAsschFcA (positive control); Lanes 2 and 6, Nuclear DNA extracted from transgenic *E.mitis*; Lanes 3 and 4, Nuclear DNA extracted from wild type *E. mitis* (negative control); M: DNA marker (DL 2000 plus marker). **c** Agarose gel electrophoresis of amplified products of PCR-based genome walking. 1–3 represents the number of PCR runs. AP2 is a random forward primer supplied in the Genome walking kit. M: DNA marker (DL2000 plus marker). **d** Integration sites of the expression cassette in the *E.mitis* genome detected using the Genome walking technique. **e** Western blotting detection of recombinant chFc protein. Lane1 and lane 2: SDS-PAGE immunoblot of transgenic and wild type *E. mitis*proteins, respectively. **f** Cellular localization patterns of chicken Fc in the sporozoites of *E. mitis* by IFA. *Scale-bar*: 10 μm

Pyrimethamine-resistant transgenic *E. mitis* was selected by adding 150 μg/mg pyrimethamine to the host, chicken diet. In the first generation, 0.2 % of the excreted oocysts expressed the YFP protein (Table 2). Subsequent passages were selected for both pyrimethamine resistance and YFP expression using pyrimethamine-containing diet and Fluorescence-Activated Cell-Sorting (FACS) of oocysts. Consequently, there has been a gradual rise in the proportion of YFP expressing oocysts in each generation up to the fifth passage (Fig. 2a), where the fluorescence-expressing population reached 93 % (Table 2).

**Table 2:** Transfection efficiency of transgenic *E. mitis* following nucleofector transfection and selection in chickens

	Percentage of fluorescent oocysts in total shed oocysts				
	1st	2nd	3rd	4th	5th
Emi.chFc	0.2 %	30 %	80 %	91 %	93 %

### Validation of stable transfection of *E. mitis*

PCR detection of YFP and chFc genes yielded products of the expected size using the genomic DNA of sporulated oocysts of transgenic *E. mitis* as the template (Fig. 2b).

The integration sites of the transfected plasmid into the *E. mitis* nuclear genome were identified by the genome walking assay from the nuclear genome. As shown in Fig. 2c and d, sequence analysis showed that the construct was integrated into the genome of *E. mitis*, with two integration sites (one in Emh_scaff1435 and the other in Emh_scaff9649). As the sites with the inserted integration have not been annotated yet, we cannot clarify the location of plasmid integration.

SDS-PAGE resolving gel of the recombinant chicken Fc protein of transgenic *E. mitis* was 26KDa, indicating the expression of chFc protein in the transgenic *E. mitis* (Fig. 2e). IFA showed that chicken Fc protein was expressed in the whole sporozoites (Fig. 2f).

### Chicken IgY Fc delivered by *E. mitis* enhances protection against wild type *E. mitis* challenge infection

**Fig. 3:**
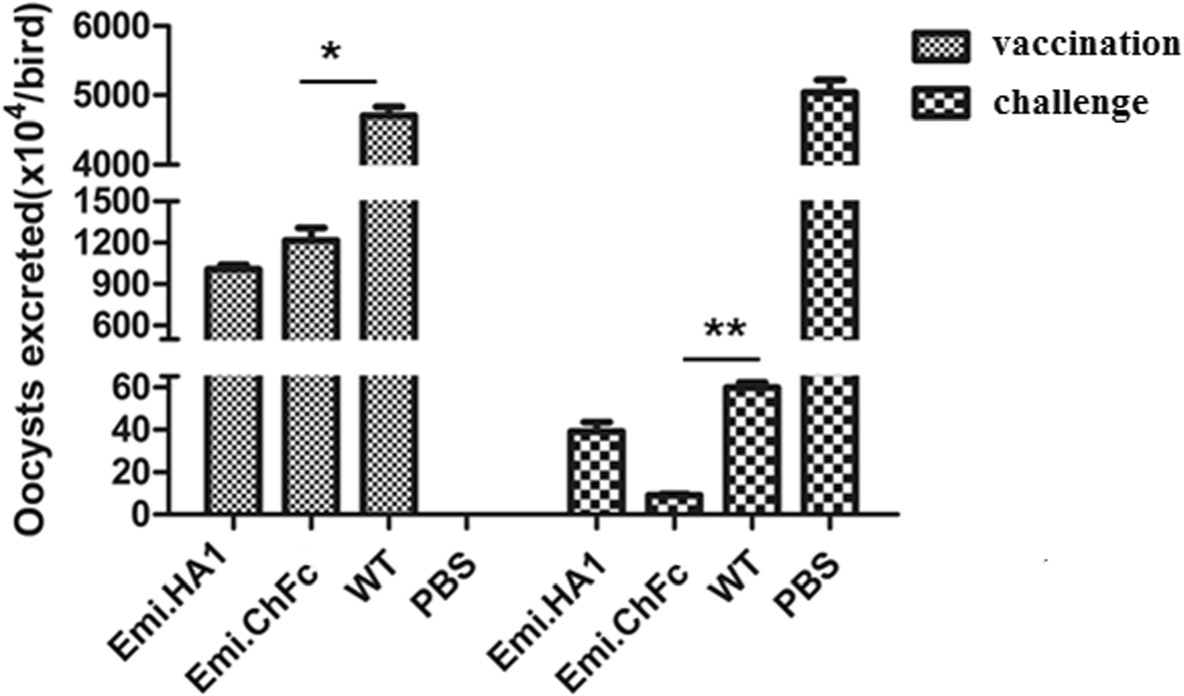
Oocysts excretion after vaccination and challenge infection in chickens. Two-week-old AA broiler chickens (*n* = 6) were vaccinated with 1000 Emi.chFc, Emi.HA1 or WT oocysts or PBS and challenged with 4 × 10^5^ WT oocysts two weeks after vaccination. Feces from each group were collected 5–8 days post-vaccination and challenge. Oocysts shedding per gram of feces was determined using a McMaster egg counting chamber. The data was pooled of four independent experiments with similar results and were expressed as the mean ± SD

Chickens were immunized by a single inoculation of the transgenic oocysts and then challenged with the wild type parasites. Oocysts excreted 5–8 days after vaccination in the Emi.chFc group (1.2 × 10^7^/bird) was significantly lower than the WT group (4.7 × 10^7^/bird) (*P* < 0.05). Two weeks after the vaccination, each bird in the Emi.chFc, WT and PBS groups was challenged with 4 × 10^5^ WT *E. mitis.* There was a significant reduction by 85 and 78 %, respectively, in oocysts shedding 5–8 days after the challenge in the Emi.chFc group (9.2 × 10^4^/bird) in comparison with the WT group (6.0 × 10^5^/bird) (*P* < 0.01) and the Emi.HA1 control group (4.0 × 10^5^/bird) while the oocysts output in the PBS group after the challenge was 5.0×107/bird (Fig. 3). The observed decline of oocyst output for birds vaccinated with Emi.chFc suggested that chicken IgY Fc delivered by *E. mitis* could enhance immunogenicity of the parasite and consequently confer better protection against *E. mitis* infection.

## Discussion

We showed that transgenic *E. mitis* expressing chicken IgY Fc fragment provoked better protective immune response than the parental *E. mitis* against *E. mitis* infection*.* Chickens immunized with *E. mitis*-vectored chicken IgY Fc fragment significantly reduced oocysts output in comparison with control groups immunized with the parental *E. mitis*, transgenic *E. mitis* expressing HA1 or PBS after challenge infection.

The mechanisms of enhanced protective immunogenicity by expressing isolated chicken IgY Fc in *E. mitis* are unknown, but the enhanced immunogenicity could be attributed to the interaction between chicken IgY Fc and its receptors which stimulated a micro-environment enhancing antigen intake. Fc receptors are key players of the immune system and link the acquired humoral immune response with innate cellular effector responses [23]. It has been postulated that mammals Fc-mediated effector function plays an important role in protective immunity to HIV in humans [24]. The avian yolk sac IgY receptor (FcRY) is the mammalian phospholipase A2 receptor orthologue and the functional counterpart of mammalian FcRn. FcRY was shown to facilitate bidirectional transcytosis and recycling of chicken IgY, suggesting it may regulate homeostasis of IgY [25]. Chicken FcRY might facilitate *E mitis* antigen presentation and thus the protective immune response of chickens. Another chicken IgY Fc receptor is CHIRAB1, which has unique high-affinity to chicken Fc [24, 26] and is mainly expressed on immature and mature B lymphocytes, and cells of the innate immune system such as monocytes, macrophages and NK cells [23]. The binding of expressed Fc to CHIRAB1 may also contribute to the enhanced immune response in chickens immunized with the chicken Fc expressing oocysts. The exact role and mechanisms of chicken IgY Fc in boosting host immunity against infections are yet to be explored.

Although live parasites vaccine for preventing coccidiosis are very successful in breeder and layer chickens, they are less justifiable economically for short-lived broilers due to the vaccination side effects and the need for repeated vaccinations with medium or low immunogenic *Eimeria* spp.. Our findings suggest that chicken Fc expressed by *E. mitis* have the potential to enhance immune response to coccidian antigens and transgenic *E. mitis* delivering chicken IgY Fc fragment could be used as an alternative, more effective coccidiosis vaccine. Further research regarding the development of transgenic *E. mitis* or *E. tenella* as a novel vaccine vector expressing chicken IgY Fc-fused exogenous antigens would be worthwhile.

## Conclusions

Transgenic *E. mitis* expressing chicken IgY Fc fragment enhanced better protective immune response against *E. mitis*. Transgenic Eimeria expressing Fc-fused exogenous antigens may be used as a novel vaccine-delivery vehicle against a wide variety of pathogens.
